# Anti-inflammatory activities of astringent persimmons (*Diospyros kaki* Thunb.) stalks of various cultivar types based on the stages of maturity in the Gyeongnam province

**DOI:** 10.1186/s12906-019-2659-5

**Published:** 2019-09-23

**Authors:** Jieun Choi, Mi Jeong Kim, Richard Komakech, Haiyoung Jung, Youngmin Kang

**Affiliations:** 1 Korean Convergence Medicine major, University of Science & Technology (UST), Daejeon, 34054 Republic of Korea; 2 Herbal Medicine Resources Research Center, Korea Institute of Oriental Medicine, 111Geonjae-ro, Naju-si, Jeollanam-do 58245 Republic of Korea; 3 Immunotherapy Research Center, Korea Research Institute of Bioscience and Biotechnology (KRIBB), Yuseong-gu, Daejeon, 34141 Republic of Korea; 4 Natural Chemotherapeutics Research Institute (NCRI), Ministry of Health, P.O. Box 4864, Kampala, Uganda

**Keywords:** Anti-inflammatory, Astringent *D. kaki*, Cultivars, Korean traditional medicine

## Abstract

**Background:**

Natural products play a significant role in human health in relation to the prevention and treatment of inflammatory conditions. One of the plants with great medicinal potentials is *Diospyros kaki* which is mainly cultivated in Asian countries including Korea, Japan, and China. Astringent *D. kaki* is a wild species with an astringent taste until they are Ripene*d. kaki* calyx is a traditional Korean medicine (TKM) made from the stalks of astringent *D. kaki* and is used in treating bed-wetting, vomiting, and hiccupping. The present study was designed to investigate the potential anti-inflammatory activities of astringent *D. kaki* stalks based on cultivar types and stages of maturity.

**Methods:**

The anti-inflammatory effects of the stalk extracts of local astringent *D. kaki* cultivar species were evaluated on RAW 264.7 cells. Cell viability was measured using a *Cell Counting Kit-8 (*CCK8) method. The anti-inflammatory effects were determined by measuring the nitric oxide (NO) concentration of the supernatant. Cellular signaling pathways were determined by quantitative polymerase chain reactions of *inducible nitric oxide synthase* (iNOS). Protein expression of iNOS and phospho-p65 was determined using western blot, and the nuclear localization of p65 was determined using confocal imaging in RAW 264.7 cells.

**Results:**

We found that the stage 1 (8–9 month) samples all showed a high percentage of tannic acid content and Gojongsi (Hamyang) stalks had the highest content. The stage 1 samples also showed the highest inhibition of NO production. Decreases in the expression of iNOS and phosphorylated p65, and in the nuclear localization of p65, were dose-dependent. All the extracts were nontoxic under 100 μg/ml concentration.

**Conclusion:**

This study provides insight into the changes in tannic acid content in astringent *D. kaki* and their anti-inflammatory effects, in relation to their stage of maturity. These results are expected to be useful in the verification of the efficacy of oriental medicine and the timing of proper harvest for medical use.

## Background

Inflammation is one of the major factors for the progression of numerous chronic diseases and disorders including diabetes, cardiovascular diseases, cancer, arthritis, obesity, autoimmune diseases, and as well as inflammatory bowel disease [1]. It involves various cells and cytokines, which provide an initial defense after infection or tissue damage by limiting damage to the affected site [2]. Macrophages are known to be involved in various host responses, such as acquired immunity and innate immunity, as well as being involved in homeostasis [3]. During the inflammatory reaction in the early stages of infection, nitric oxide (NO) and cytokine are produced and play an important role in biological defense mechanisms [4]. Although inflammation can be treated by use of synthetic drugs, some of the anti-inflammatory drugs such as anticytokine have some negative effects of blocking the activity of various kinases hence resulting in a significant decrease in host defence toward infections [1]. Due to some of these side effects and health problems of existing anti-inflammatory therapies, the use of natural anti-inflammatory supplements are becoming more popular [1]. Indeed, natural products play a significant role in human health in relation to the prevention and treatment of inflammatory conditions [5–7]. Consequently, the demand for natural effective anti-inflammatory agents to serve as safe and effective therapeutic agents has gained increasing attention [8]. The anti- inflammatory potential of plants are attributed to the presence of secondary metabolites such as saponins, glycosides, and phenolic compounds including tannins and flavonoids [9]. Additionally, these phytochemicals such as tannin which is a phenol compound has various physiological functions such as anti-mutagenic and anti-cancer effects [10, 11]. Tannic acid has a particularly high molecular weight and contains many phenolic hydroxyl groups, which cause its bitter taste [12]. In fact, the taste is due to the dehydration caused by the interaction between the tannin and the proteins on the surface of the tongue [13]. A study by Robbins et al., [14] reported that tannins are one of the secondary metabolites that plants use in defensive mechanisms against large herbivores.

Persimmon (*Diospyros kaki* Thumb.) Family Ebenaceae is one of the plants with the potential to ameliorate *inflammatory* responses owing to its ability to scavenge free radicals [15]. This important plant has been cultivated for many years in Asian counties, especially in Korea, China, and Japan [16]. In South Korea specifically, most of the *D. kaki* is distributed within the inland provinces of Gapyeong (Gyeonggi), Byeonghwa (Gyeongbuk), and Jecheon (Chungbuk), and along the north and east coasts [17]. *D. kaki* cultivars such as Bansi (Chungdo), Danseongsi, and Wolhasi have been made more suitable for cultivation in local temperature and soil conditions [18]. In fact, studies have shown that *D. kaki* has a lower tolerance for cold than other fruit trees [19]. *D. kaki* can be classified as sweet varieties and astringent varieties [20]. Astringent *D. kaki* is edible after reducing their astringency by ripening or by artificially removing their high water-soluble tannin content [21, 22]. *D. kaki* flesh has been eaten for centuries and in recent times the fruits have been processed into jam and vinegar [23–25]. The dried leaves are also used as green tea in many communities [26]. The medicinal potential of *D. kaki* is attributed to the various bioactive compounds contained in it such as tannins, flavonoids, terpenoids, steroids, carotenoids, gallic acid, and protocatechuic acid [27]. Many phenolic compounds are known to be anti-oxidative, anti-inflammatory, anti-carcinogenic, anti-mutagenic, and cardioprotective [28, 29]. Clinical pharmacokinetics and the efficacy of the stalks of *D. kaki* are well reported in ancient literature [30]. Kaki calyx is a traditional Korean medicine (TKM) made from the calyx attached to the fruit of *D. kaki* [31]. In the 4th edition of the Korean Herbal Pharmacopoeia, Kaki calyx is defined as the calyx attached to the matured or ripened fruit of *D. kaki*. Studies have showed that *D. kaki* has positive effects on blood vessels, lung strengthening and also important for the alleviation of bronchial diseases [32, 33]. In TKM, the stalks of astringent *D. kaki* have been used in the treatment of flu, atherosclerosis, and heart diseases [32, 34]. Additionally, *D. kaki* stalks have also been used in TKM for the treatment of hiccups [32, 34]; a condition in which inflammation is known to be one of the causes [35].

Despite the fact that a number of studies have been conducted to develop therapeutic targets from natural sources that are intended for suppression of various chronic inflammations associated diseases [1], no study has been conducted on the anti-inflammatory activities of astringent *D. kaki* stalks of various cultivar types based on the stages of maturity from the Gyeongnam province. This study therefore was conducted to determine the optimal harvesting time of several selected cultivars of astringent *D. kaki* from various regions, based on their efficacy as herbal medicines, including their tannic acid content and anti-inflammatory effects. In addition, understanding the variations in tannic acid concentration and anti-inflammatory potential of *D. kaki* could be important for the industry and traditional oriental medicine development.

## Methods

### Collection of astringent *D. kaki*

In this study, three local astringent *D. kaki* cultivars (Gojongsi, Danseongsi, and Bansi) were collected from the Hamyang, Hamyang, and Miryang regions respectively, during three different seasons (stage 1, August 19th; stage 2, October 14th; and stage 3, January 20th). The specimens were identified at Korea Forest Environment Research Institute at Gyeongsangnam, Korea and were deposited at a publicly available herbarium in Korea Institute of Oriental medicine and given voucher specimen numbers KIOM201501015023- KIOM201501015025. The astringent *D. kaki* samples were collected according to regional characteristics and maturity and the stalks cut and classified as immature stage (collected from August to September), mature stage (collected from October to November), and dried stage (collected from December to January).

### Extraction from stalks of astringent *D. kaki*

The 9 samples used in this experiment were 3 samples of immature stalks, 3 samples of mature stalks, and 3 samples of dry stalks obtained from Hamyang Gojongsi, Hamyang Danseongsi, and Miryang Bansi. The ethanol and methanol reagents used for the extraction process were purchased as high-performance liquid chromatography (HPLC)-grade (JT Baker Inc., Phillipsburg, NJ, USA). The solvents and samples were filtered with a 0.2 μm membrane filter (PALL Corporation, Ann Arbor, MI, USA) before use. For each of the 9 samples, 10 g of the dried ground sample was extracted using maceration method with 250 mL 70% ethanol for 72 h. The extracts were filtered using filter paper (ADVANTEC, 110 mm, Toyo Robni kaisha, Japan) and concentrated under a vacuum reduced pressure at 40 °C, RPM 70 using EYELA N-1200B (Tokyo Rikakikai Co. Ltd., Japan) efficient rotary evaporator. The concentrated extract was then vacuum dried. 50 mg/mL of solution was then obtained by separately dissolving each of the extracts from the different samples in 80% methanol. For HPLC analysis, samples were filtered using a 0.45 μm membrane filter (PALL Corporation, Ann Arbor, MI, USA) prior to injection and analysis.

### Analysis of tannic acid contents using HPLC

An HPLC system (Alliance® HPLC, Waters Corporation, Manchester, UK) was used in this study. The column was a Phenomenex Luna C18 (2) (250 × 4.6 mm, 5 μm), the column temperature was 40 °C, and the sample temperature was 25 °C. The mobile phase consisting of HPLC grade water with 0.1% acid and HPLC grade Methanol with 0.1% acid was used. The calibration curves were analyzed using 5 concentrations (25, 50, 100, 150, and 200 μg/mL) of tannic acid standard (Sigma-Aldrich Co., Inc. FLUKA, Trace SELECT, USA). The flow rate of the mobile phase was set to 1.0 mL/min, the injection amount of the standard product was set to 10 μL, while the injection amount of the 9 samples was set to 20 μL. The analysis time for all samples was set to 30 min in total. The analytical wavelength was measured at 190 to 400 nm and analyzed at 220 nm. The UV spectrum of each component was measured. All HPLC-related experiments were repeated three times and the content analysis was completed with an average value.

### Preparation of RAW 264.7 cells and cell viability assay (CCK-8 assay)

RAW 264.7 macrophages were cultured in RPMI 1640 medium (WelGENE, Daegu, Korea) containing 10% fetal bovine serum (FBS) and 1% antibiotics, at 37 °C in a 5% CO_2_ atmosphere. RAW 264.7 macrophages were treated with various stalk extract concentrations (0, 10, 50, 100, 200, 500, and 1000 μg/ml) with different maturities for 24 h. The proliferation of RAW 264.7 macrophage cells on the substrates was determined by cell counting kit assay (CCK-8, Dojindo, Kumamoto, Japan). All samples were placed in a 96-well plate and seeded with a density of 5 × 10^4^ cells/well. Absorbance was measured at 450 nm using an ELISA plate reader.

### NO assay and immunoblotting using western analysis

The NO level produced from the RAW 264.7 cell lines was determined using a commercially available NO detection kit (iNtRON, Inc., Seoul, Korea). RAW 264.7 cells were pretreated with stalk extracts (at 0, 10, 20, 50, and 100 μg/ml) for 1 h, followed by incubation with lipopolysaccharides (LPS, 1 μg/ml) for 24 h. All samples were placed in a 48-well plate and seeded with a density of 1 × 10^5^ cells/well. After incubating for 24 h, 100 μL of supernatant was collected and added to wells in triplicate; 50 μL of N1 buffer was added to each well, and the plate was incubated at room temperature for 10 min. Then, 50 μl of N2 buffer was added and the final reaction was incubated at room temperature for 10 min. The absorbance was measured at 540 nm using a multi-plate reader (Lambda Bio-20; Beckman Coulter, Inc., Brea, CA, USA).

The nitrite concentration of the supernatant was determined using a nitrite standard curve. For the western blot analysis, RAW 264.7 cells were pretreated with stalk extracts (at 0, 10, 20, 50, and 100 μg/ml) for 1 h, followed by incubation with LPS (1 μg/ml) for 24 h. All samples were placed in a 6-well plate and seeded with a density of 1 × 10^6^ cells/well. Cells were harvested with ice-cold phosphate-buffered saline (PBS), and lysed in lysis buffer (50 mM Tris-HCl, pH 7.5, 150 mM NaCl, 1% v/v IGEPAL® CA-630, 1 mM PMSF, 1 mM sodium fluoride, and 10 μg/ml aprotinin and leupeptin). The cell lysates were collected by centrifuging at 13,000 rpm for 15 min at 4 °C. The protein concentration of the collected supernatants was determined using a BCA™ protein assay kit (Pierce, Rockford, IL, USA). All lysates were loaded with the same amount of protein on SDS-PAGE gel. Proteins were then transferred to a PVDF membrane (Millipore, Bedford, MA, USA). After blocking with 5% w/v skimmed milk in 1xPBST at 27 °C for 40 min, the membrane was incubated for 2 h with specific primary antibodies (1/1000 dilution in 5% w/v skimmed milk in PBST) and then incubated with the HRP-conjugated secondary antibodies (1/2500 dilution in 1x PBST) for 1 h at room temperature.

### RNA isolation and quantitative real-time PCR

Total RNA was extracted from the RAW 264.7 cell lines using an RNeasy Mini Kit (Qiagen, Gaithersburg, MD, USA) according to the manufacturer’s instructions. Cells were plated at a density of 1 × 10^6^ cells/well in a 6-well plate, and pretreated with stalk extracts at concentrations of 0, 10, 50, and 100 μg/ml for 1 h. Thereafter, cells were stimulated with LPS (1 μg/ml) for 24 h. After 24 h of incubation, the cells were washed with cold PBS and collected. Total RNAs were reverse-transcribed to first strand cDNAs using a ReverTra Ace® qPCR RT Kit (Toyobo Co, Osaka, Japan) and analyzed by real time PCR (Takara Bio Inc., Shiga, Japan) using an SYBR Green PCR Master Mix (Takara Bio Inc., Shiga, Japan) with specific primers. The mRNA expression level was calculated using GAPDH as a control. The primer sequences were as follows: inducible nitric oxide synthase (iNOS), forward 5′- GGCAGCCTGTGAGACCTTTG-3′ and reverse 5′- GCATTGGAAGTGAAGCGTTTC-3′; GAPDH, forward 5′-ATGCCTCCTGCACCACCA-3′ and reverse 5′- CCATCACGCCACAGTTTCC-3′.

### Confocal microscopy for p65 localization

The effect of the stalk extracts on the nuclear translocation of nuclear factor κB (NF-κB) p65 was evaluated by immunofluorescence. For these experiments, RAW 264.7 cells were pretreated with 100 μg/ml stalk extracts for 1 h and harvested by treatment with LPS (1 μg/ml) for 30 min. Cells were plated on round glass cover slips in 12-well plates for 10 min. The samples were then washed with cold PBS and fixed in 4% formaldehyde for 15 min at room temperature, followed by permeabilization with 0.2% Triton X-100 in PBS for 15 min at 4 °C. Cells were blocked for 1 h with 5% BSA in PBS and incubated with anti-p65 primary antibody at 4 °C overnight. After three washes with PBS, the cells were incubated with Alexa Fluor 546-conjugated goat-anti rabbit IgG (Invitrogen, Carlsbad, California, USA) for 1 h at room temperature, and then washed again with PBS. The images were captured using an LSM510 confocal microscope (Carl Zeiss, Gottingen, Germany).

### Statistical analysis

Data collected from each group (experimental and control) were expressed as mean ± SD. Unpaired t-tests (in the Prism program, Graph Pad Software, San Diego, CA; two-tails, *P* < 0.05) were used to analyze the differences between experimental and control groups.

## Results

### Analysis of tannic acid contents using HPLC

**Fig. 1:**
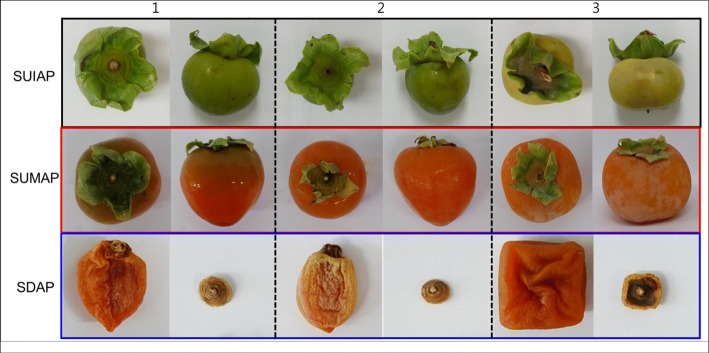
The appearance of astringent *D. kaki* from three different areas of Gojongsi in Hamyang, Danseongsi in Hamyang, and Bansi in Miryang obtained at different seasons. 1: Stalk of undried immature astringent *D. kaki* (SUIAP) in Hamyang (Gojongsi), Hamyang (Danseongsi), and Miryang (Bansi). 2: Stalk of undried mature astringent *D. kaki* (SUMAP) in Hamyang (Gojongsi), Hamyang (Danseongsi), and Miryang (Bansi). 3: Stalk of dried astringent *D. kaki* (SDAP) in Hamyang (Gojongsi), Hamyang (Danseongsi), and Miryang (Bansi)

**Fig. 2:**
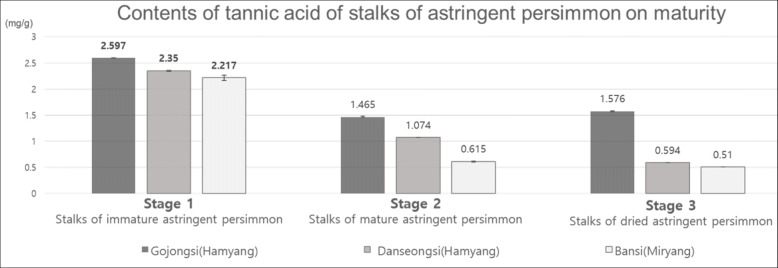
Analysis of tannic acid contents. Contents of tannic acid of stalks from different stages of maturity in astringent *D. kaki*. Summarized data was indicated Fig. 2 from the previously published paper from Choi et al. (2017) JALS 51(3):49–60

Extracts from the different stalks (Fig. 1) were analyzed by HPLC to determine the contents of tannic acid. The retention time of all the samples and of standard tannic acid was found to be 6 min. The HPLC analysis of the contents of tannic acid in relation to maturity showed that tannic acid content was higher in the immature astringent *D. kaki* than in the mature or dried ones (Fig. 2). As shown in Fig. 2, stalks of undried immature astringent *D. kaki* from Hamyang Gojongsi had the highest content of tannic acid (2.597 mg/g) followed by Hamyang Danseongsi (2.350 mg/g), and then Miryang Bansi (2.217 mg/g). The content of tannic acid tended to decrease according to the degree of ripeness, with the immature *D. kaki* stalks having highest tannic acid concentration and the least found in the dried stalks. The content of tannic acid in Miryang Bansi stalks decreased more rapidly between the immature and mature stage (from 2.217 mg/ml to 0.615 mg/ml), compared to that in Hamyang Gojongsi and Hamyang Danseongsi stalks.

### Effects of stalk extracts on cell viability

**Fig. 3:**
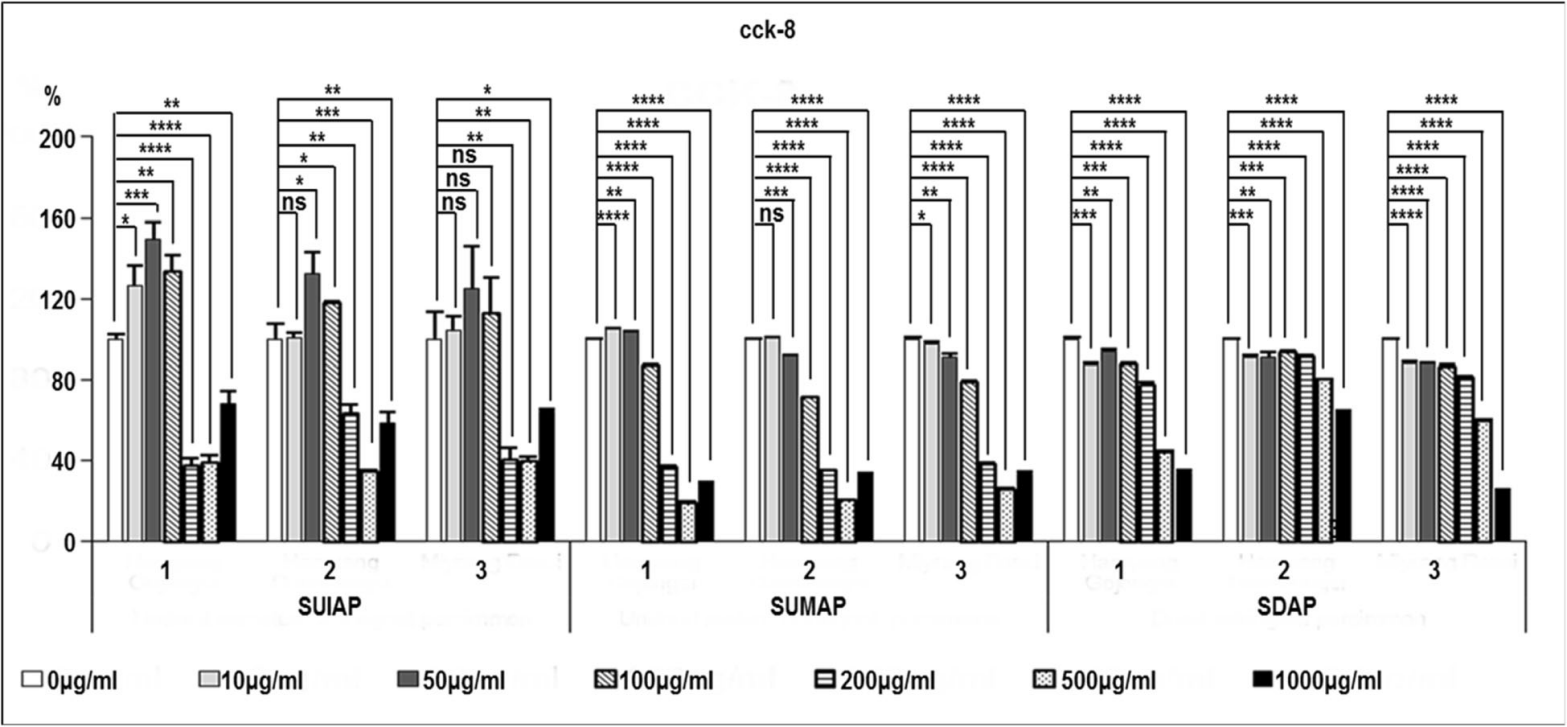
Effects of various stalk extracts on cell viability in RAW 264.7 macrophages cells. Cells were treated with concentrations (0, 10, 50, 100, 200, 500, 1000 μg/ml) of various stalk extract for 24 h. Cell viability was measured using the CCK-8 assay. The results are presented as the mean ± SD of at least three independent experiments. *P < 0.05, **P < 0.01, *** *P* < 0.001, **** *P* < 0.0001

The results indicate that treatment with concentrations of 10, 50, and 100 μg/ml of undried immature astringent *D. kaki* stalk extract promoted the viability of RAW 264.7 cells compared to control cells, while higher concentrations (200, 500, 1000 μg/ml) had significant negative effects on the viability of the cells (Fig. 3).

### Effects of stalk extracts on the production of NO in LPS-stimulated RAW 264.7 cells

**Fig. 4:**
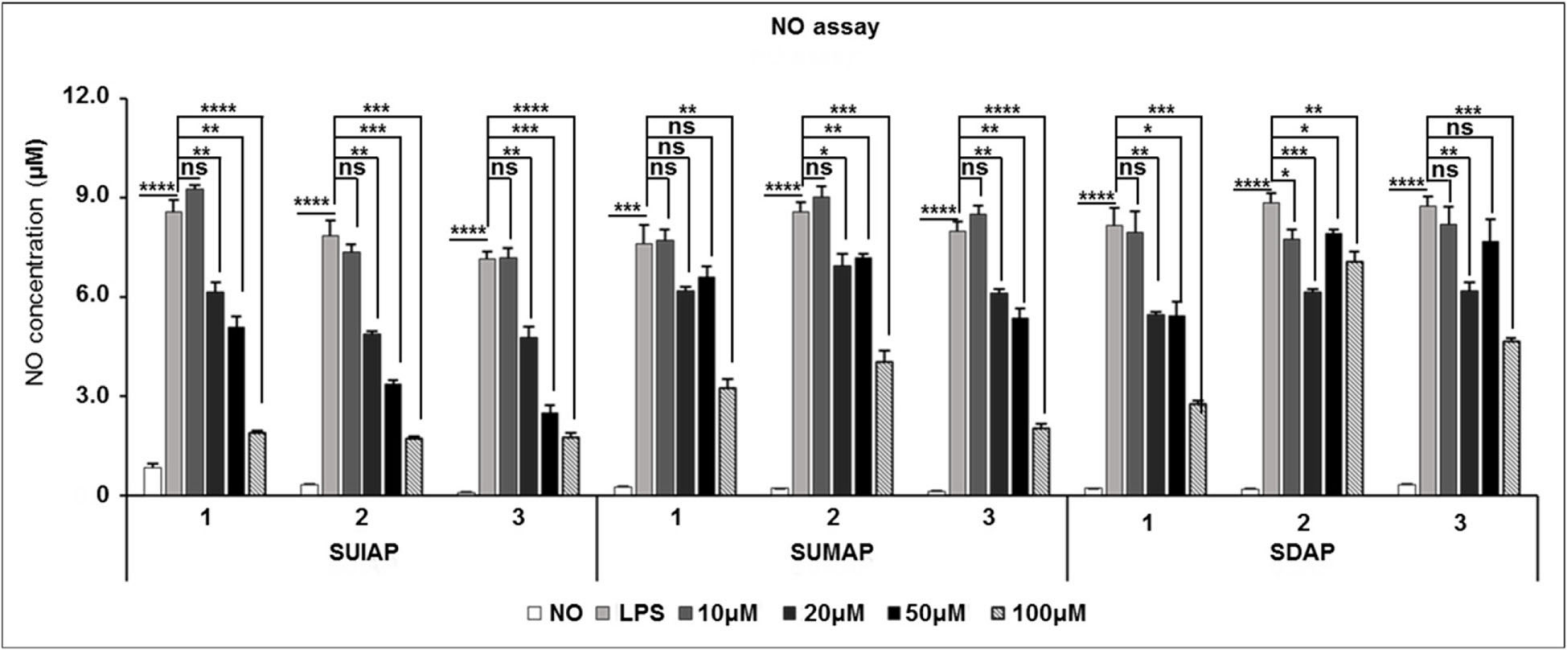
Inhibitory effects of various stalk extracts on NO production in LPS-induced RAW 264.7 cells. Cells were pretreated with various concentrations (0, 10, 20, 50, 100 μg/ml) of stalk extracts for 1 h and then LPS (1 μg/ml) was added and incubated for 24 h. After 24 h, the nitrite concentrations in medium were determined by NO Detection Kit. The results are presented as the mean ± SD of at least three independent experiments. *P < 0.05, **P < 0.01, *** *P* < 0.001, **** *P* < 0.0001

All the sample extracts showed inhibitory effects on the production of NO in LPS-activated RAW 264.7 macrophages. Cells treated with extracts from the stalks of immature astringent *D. kaki* showed a greater reduction in NO production compared to those treated with more mature stalk extracts (Fig. 4).

### Effects of stalk extracts on iNOS expression in LPS-stimulated RAW 264.7 cells

**Fig. 5:**
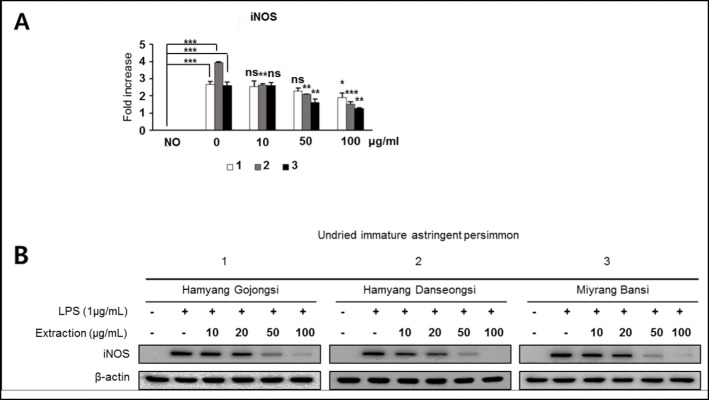
Inhibitory effect of stalk extracts on LPS-stimulated upregulation of iNOS expression in RAW 264.7 macrophages. The cells were pre-treated with different concentration (0, 10, 50, 100 μg/ml) of stalk extracts for 1 h followed by stimulated with LPS (1 μg/ml) for 24 h. **a** The levels of iNOS were quantified by RT-qPCR and normalized to GAPDH. **b** The cells were pretreated with stalk extracts at the indicated concentrations for 1 h before incubation with LPS (1 μg/ml) for 24 h. The protein expression of iNOS and β-actin were assessed by western blot analysis. The results are presented as the mean ± SD of at least three independent experiments. *P < 0.05, **P < 0.01, ***P < 0.001

The expression of iNOS was strongly inhibited by extract treatments at 100 μg/ml (Fig. 5a). The stalk extracts from immature astringent *D. kaki* decreased the protein expression of iNOS, in a dose-dependent manner (Fig. 5b). These results strongly suggest that the inhibitory effects of the extracts on LPS-induced NO production are caused by the suppression of iNOS expression, as the extracts regulated the expression of the iNOS enzyme at the mRNA, as well as the protein, level.

### Effects of stalk extracts on the activation of NF-κB in LPS-stimulated RAW 264.7 cells

**Fig. 6:**
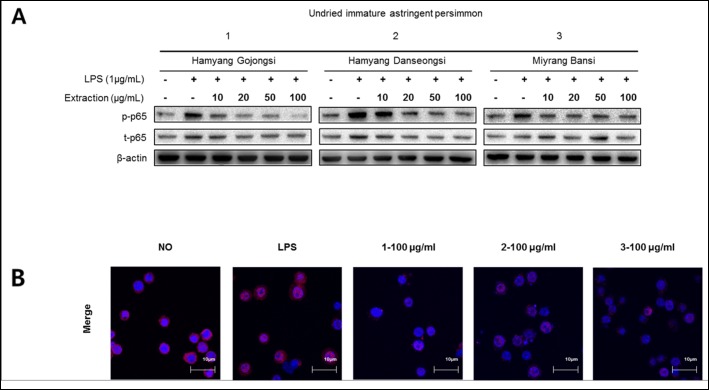
Inhibitory effects of stalk extracts on LPS-induced NF-kB activation. **a** Inhibitory effect of stalk extracts on LPS-induced p65 phosphorylation in RAW 264.7 macrophages. The cells were pretreated with stalk extracts at the indicated concentrations for 1 h before incubation with LPS (1 μg/ml) for 1 h. The protein expression of p-p65 and β-actin were assessed by western blot analysis. **b** Inhibitory effect of stalk extracts on LPS-induced p65 nuclear translocation in RAW 264.7 macrophages. Cells were pretreated with stalk extracts (100 μg/ml) for 1 h followed by stimulation with LPS (1 μg/ml) for 30mins. Samples were stained by anti-p65 antibody, Alexa Fluor 546 goat anti-rabbit antibody and DAPI then determined by confocal microscopy analysis

As shown in Fig. 6a, phosphorylated p65 was significantly inhibited by increasing the concentration of the extracts. The positive control, LPS-stimulated RAW 264.7 cells, showed dramatic increases in the translocation of p65 into the nucleus, however LPS stimulated NF-κB nuclear translocation was markedly suppressed after pre-treatment in stalk extract (Fig. 6b). These results suggest that stalk extract inhibits the activation of NF-κB by inhibiting the phosphorylation and nuclear translocation of p65.

## Discussion

*D. kaki* has various medicinally bioactive compounds such as carotenoids, tannins, flavonoids, sugars, hydrocarbons, lipids, hydrocarbons, aromatics, terpenoids and steroids. The astringent *D. kaki* is a wild species and have an astringent taste until fully ripened, at which stage the tannin content is completely transformed into an insoluble structure. Astringent *D. kaki* containing water-soluble tannin components have a bitter taste and the tannic acid has been shown to have anti-aging, anticancer and herbivory defense effects [13, 20, 25]. We obtained extracts from the stalks of astringent *D. kaki* and analyzed the contents of tannic acid according to their level of maturity. The tannic acid contents of three kinds of astringent *D. kaki* at different stages of maturity indicated that all the immature stalks had higher tannic acid content than the mature and dried stalks. The results of this study are consistent with those of a study by Jeong [18, 34], showing that the concentration of tannic acid in *D. kaki* decreases as the astringency is removed during the drying process. *D. kaki* show a three-stage S-shaped growth curve and their sugar content has been reported to be significantly affected by sunshine, especially after October [36]. In this study, the useful component content was significantly different depending on the growth stage of the *D. kaki*. Del Bubba et al. [37] found that the contents of soluble tannic acid in cultivated Rojo Brillante and Kaki Topo, increased from July to November but decreased rapidly in December, while the content of sugar increased from September. Additionally, various weather factors including temperature, monthly precipitation, duration of sunshine, and diurnal range, have been observed to influence *D. kaki* fruit weight and quality [17]. Of the nine samples analyzed, the stalks of immature astringent *D. kaki* from Hamyang Gojongsi had the highest content of tannic acid (2.597 mg/g). The contents of tannic acid from Myrang Bansi samples decreased considerably from 2.217 mg/g to 0.615 mg/g in the immature and mature stages, respectively. Lee [38] also reported that the phenol contents of Cheongdo Bansi fruits rapidly decreased during July and August and during September and October. In this study, the tannic acid contents of astringent *D. kaki* stalks from the three different regions showed substantial differences in tannic acid content at the mature stage. Previous analysis of the tannic acid contents of astringent *D. kaki* from three different regions (Sancheong Gojongsi, Sancheong Danseongsi, and Miryang Bansi) showed that tannic acid content was 30% higher in undried *D. kaki* than in dried ones [39]. The change in tannic acid contents of three cultivars from the Gyeongsangnam-do province in 2015 and 2016 showed similar tendencies. To increase the utilization of astringent *D. kaki*, we investigated tannic acid content according to maturity and established it’s important to harvest astringent *D. kaki* when they are fully matured; at a time when the tannic acid is at its lowest. However, further analysis needs to be performed across various seasons for comprehensive results. In addition to the effects due to the variation in environmental conditions, the astringency of *D. kaki* may also be attributed to its genetic factors [40]. *Diospyros kaki* is known to accumulate a large amount of proanthocyanidins in its fruit, resulting in its astringent taste [41]. The transcriptional regulation of proanthocyanidin biosynthesis pathways may be one of the many factors attributed to the varying level of astringency in the different cultivars of persimmon fruits [40, 42]. The Myb transcription factor (DkMYB4) and bHLH transcription factor (DkMYC1) which forms a protein complex with each other for proanthocyanidins regulation may have a central role in the differential expression of proanthocyanidins in the different *D. kaki* cultivars [41] and hence the variation in the level of astringency in them. Furthermore, a WD40-repeat protein from persimmon has been observed to interact with the regulators of proanthocyanidin biosynthesis DkMYB2 and DkMYB4 to form a MYB-bHLH-WD40 complexes in persimmon to regulate proanthocyanidin accumulation in fruits [42].

The stalks of astringent *D. kaki* have been used as traditional medicine in Korea (known as Kaki calyx) and are used for the treatment of bed-wetting, vomiting and hiccups. Studies have also shown that *D. kaki* has anti-inflammatory effects. Macrophages are critical for the development of inflammatory reactions, as they produce various pre-inflammatory mediators. NO plays an important role in biological defense mechanisms [4] but excessive production of NO results in the development of inflammatory diseases such as rheumatoid arthritis and autoimmune disorders [43]. Therefore, to inhibit NO production is a major target for anti-inflammatory treatments. We therefore investigated whether extracts from the stalks of astringent *D. kaki* blocked NO production and iNOS protein expression in a LPS-stimulated inflammatory condition. Immature astringent *D. kaki* was found to be the most effective and acted dose-dependently (Fig. 4). As shown in Fig. 5, extracts obtained from the stalks of immature astringent *D. kaki* decreased the protein expression of iNOS in a dose-dependent manner. In most types of cells NF-κB is an important transcriptional factor related to inflammation. NF-κB is a family of dimeric molecules with pro- and anti-inflammatory properties. In mammals, the NF-κB family comprises five proteins: NF-κB1 (p50/105), NF-κB2 (p52/100), Rel A (p65), Rel B and c-Rel [34, 44]. Of these, the p65/p50 heterodimer is the most predominant pro-inflammatory complex and p65 nuclear translocation and DNA binding is typically defined as the activation of the NF-κB pathway [45]. Upon activation by external stimuli, such as LPS, the IκB protein is phosphorylated, degraded, and translocated into the nucleus. Therefore, degradation of IκB makes NF-κB free to translocate to the nucleus, where it regulates gene transcription. NF-κB activation leads to the transcription of pro-inflammatory mediators and cytokines such as iNOS, COX-2 and NO [46]. We found that stalk extracts suppressed NO production and decreased the expression of iNOS at transcriptional and post-transcriptional levels, as well as reducing p65 nuclear translocation in LPS-stimulated RAW 264.7 cells. These results indicate that stalk extracts inhibit the expression of iNOS through inactivation of NF-κB by reducing p65 phosphorylation. The inhibition of the NF-κB pathway in RAW 264.7 macrophages down-regulated the pro-inflammatory mediators hence exhibiting the anti-inflammatory effects of the stalks of immature astringent *D. kaki*. Apart from the stalks, previous study also showed that the fruits of *D. kaki* has the potential to ameliorate *inflammatory* responses owing to its ability to scavenge free radicals [15, 34]. Consequently, the anti-inflammatory potential of the stalks of immature astringent *D. kaki* can be exploited for use in treatment of disease conditions.

## Conclusion

This study investigated the tannic acid content, and anti-inflammatory activities of astringent *D. kaki* (*Diospyros kaki* Thunb.) stalks in the Gyeongnam province, based on cultivar types and stages of maturity, to suggest the optimal harvesting time based on their efficacy as an herbal medicine. All the samples analyzed were found to contain tannic acid and the stage 1 (8–9 month old) samples had the highest percentage of tannic acid content, especially those from Gojongsi (Hamyang). In addition, the anti-inflammatory effects of stalk extracts were confirmed and their effects were found to decrease according to maturity. This study provides basic information on how the tannic acid content of astringent *D. kaki* stalks changes at different levels of maturity. The anti-inflammatory effects of these extracts are expected to be helpful in the verification of the efficacy of oriental medicine. We suggest that the optimum harvest times for medical and non-medical use of astringent *D. kaki* are 8–9 months, although further research is necessary to collaborate these findings.

## Data Availability

The raw data has been deposited at a publicly available repository at Standard herbal Resources Center of Korean Institute of Oriental Medicine and is available upon reasonable request to the authors.

## Abbreviations

CCK8: Cell Counting Kit-8
COX-2: Cyclooxygenase-2
DNA: Deoxyribonucleic acid
HPLC: High-performance liquid chromatography
INOS: Inducible nitric oxide synthase
LPS: Lipopolysaccharide
NF-Κb: Nuclear factor-kappa B
NO: Nitric oxide
PCR: Polymerase chain reaction
RNA: Ribonucleic acid
TKM: Traditional Korean medicine
